# Bacteriological analysis based on disease severity and clinical characteristics in patients with deep neck space abscess

**DOI:** 10.1186/s12879-022-07259-9

**Published:** 2022-03-23

**Authors:** Wenxiang Gao, Yu Lin, Huijun Yue, Weixiong Chen, Tianrun Liu, Jin Ye, Qian Cai, Fei Ye, Long He, Xingqiang Xie, Guoping Xiong, Jianhui Wu, Bin Wang, Weiping Wen, Wenbin Lei

**Affiliations:** 1 Otorhinolaryngology Hospital, First Affiliated Hospital, Sun Yat-Sen University, 58 Zhongshan 2nd Road, Guangzhou, Guangdong People’s Republic of China; 2 Department of Otorhinolaryngology-Head and Neck Surgery, First People’s Hospital of Foshan, Foshan, Guangdong People’s Republic of China; 3 Department of Otorhinolaryngology-Head and Neck Surgery, Sixth Affiliated Hospital of Sun Yat-Sen University, Guangzhou, Guangdong People’s Republic of China; 4 Department of Otorhinolaryngology-Head and Neck Surgery, Third Affiliated Hospital of Sun Yat-Sen University, Guangzhou, Guangdong People’s Republic of China; 5 Department of Otorhinolaryngology-Head and Neck Surgery, Sun Yat-Sen Memorial Hospital of Sun Yat-Sen University, Guangzhou, Guangdong People’s Republic of China; 6 Department of Otorhinolaryngology-Head and Neck Surgery, Zhongshan People’s Hospital, Zhongshan, Guangdong People’s Republic of China; 7 Department of Otorhinolaryngology-Head and Neck Surgery, First People’s Hospital of Guangzhou, Guangzhou, Guangdong People’s Republic of China; 8 Department of Otorhinolaryngology-Head and Neck Surgery, First People’s Hospital of Zhaoqing, Zhaoqing, Guangdong People’s Republic of China; 9 Department of Otorhinolaryngology-Head and Neck Surgery, Jiangmen Central Hospital Affiliated Jiangmen Hospital of Sun Yat-Sen University, Jiangmen, Guangdong People’s Republic of China

**Keywords:** Deep neck space abscess, Bacterial analysis, Disease severity, Clinical characteristics, Gram-staining strains

## Abstract

**Background:**

Deep neck space abscess (DNSA) is a serious infection in the head and neck. Antibiotic therapy is an important treatment in patients with DNSA. However, the results of bacterial culture need at least 48 h, and the positive rate is only 30–50%, indicating that the use of empiric antibiotic treatment for most patients with DNSA should at least 48 h or even throughout the whole course of treatment. Thus, how to use empiric antibiotics has always been a problem for clinicians. This study analyzed the distribution of bacteria based on disease severity and clinical characteristics of DNSA patients, and provides bacteriological guidance for the empiric use of antibiotics.

**Methods:**

We analyzed 433 patients with DNSA who were diagnosed and treated at nine medical centers in Guangdong Province between January 1, 2015, and December 31, 2020. A nomogram for disease severity (mild/severe) was constructed using least absolute shrinkage and selection operator–logistic regression analysis. Clinical characteristics for the Gram reaction of the strain were identified using multivariate analyses.

**Results:**

92 (21.2%) patients developed life-threatening complications. The nomogram for disease severity comprised of seven predictors. The area under the receiver operating characteristic curves of the nomogram in the training and validation cohorts were 0.951 and 0.931, respectively. In the mild cases, 43.2% (101/234) had positive culture results (49% for Gram-positive and 51% for Gram-negative strains). The positive rate of cultures in the patients with severe disease was 63% (58/92, 37.9% for Gram-positive, and 62.1% for Gram-negative strains). Diabetes mellitus was an independent predictor of Gram-negative strains in the mild disease group, whereas gas formation and trismus were independent predictors of Gram-positive strains in the severe disease group. The positivity rate of multidrug-resistant strains was higher in the severe disease group (12.1%) than in the mild disease group (1.0%) (*P* < 0.001). Metagenomic sequencing was helpful for the bacteriological diagnosis of DNSA by identifying anaerobic strains (83.3%).

**Conclusion:**

We established a DNSA clinical severity prediction model and found some predictors for the type of Gram-staining strains in different disease severity cases. These results can help clinicians in effectively choosing an empiric antibiotic treatment.

## Introduction

Deep neck space abscess (DNSA) is defined as a collection of pus in the fascial planes and potential spaces of the neck, such as parapharyngeal, retropharyngeal, prevertebral, submandibular, carotid, visceral, and submental spaces, except for the peritonsillar space. Because of its acute onset, hidden location, and rapid progress, delayed treatment can result in life-threatening complications such as airway obstruction, descending mediastinitis, and sepsis [1]. Thus, DNSA has been recognized globally as a serious public health disease.

The administration of antibiotics is an essential part of the treatment strategy for patients with DNSA. Generally, antibiotics should be selected based on bacterial culture and susceptibility test results. However, according to previous reports, the positivity rate of bacterial culture is approximately 30%–50%, and at least 48 h are required to attain the results, indicating that most patients must rely on empiric antibiotic treatment within 48 h or even throughout the treatment course [2–4]. Therefore, the selection of effective empiric antibiotics is a conundrum that has always plagued clinicians.

Considering that DNSA is typically a polymicrobial infection (e.g., coinfection with aerobic and anaerobic bacteria), most clinicians recommend the use of broad-spectrum antibiotics as empiric treatment [2, 5–7]. However, using a narrow-spectrum antibiotic would reduce changes to the normal flora and adverse events, thereby limiting the overgrowth of resistant nosocomial organisms, avoiding the potential toxicity of multiple-drug regimens, and reducing costs, which is in line with the goals of the antimicrobial stewardship program (ASP) in China [8, 9]. But so far there are no guidelines in clinical settings on the use of empiric antibiotics before bacterial culture and drug susceptibility results are available. Bakir et al. suggested that empiric antibiotic treatments should be selected based on disease severity in patients with DNSA [10].

Therefore, the rational use of empiric antibiotics in DNSA patients must be considered in combination with the potential types of bacteria and disease severity. In this study, we established a DNSA clinical severity prediction model and further predicted the types of potential infecting bacteria (Gram-staining positive/negative) based on relevant clinical characteristics, aiming to provide clinical knowledge with certain guidelines for the rational selection of empiric antibiotics in clinical practice.

## Methods

### Study design and participants

This was a retrospective, multicenter study that included patients with DNSA from nine medical centers in five different cities representing the Pearl River Delta region, Guangdong Province, from January 1, 2015, to December 31, 2020. Patients who met the diagnosis of DNSA (retropharyngeal, parapharyngeal, and submandibular abscesses) (International Classification of Diseases, Tenth Revision, Clinical Modifications coding J39.002, J39.004, and L02.051) were included in this study. Patients with the following conditions were excluded: (1) benign or malignant tumors, (2) history of chemoradiotherapy, (3) infections secondary to surgical neck trauma, and (4) those who did not accept treatment.

To develop a prediction model for disease severity (mild/severe), the included patients were divided into training and validation datasets. Those who were treated between January 1, 2015, and December 31, 2019, were assigned to the training cohort. The patients who were treated from January 1, 2020, to December 31, 2020, formed the validation cohort, which was established consecutively based on the same standards as the training cohort.

### Data collection

All data were collected at admission, including demographic characteristics (age > 18 years, sex, smoking, and alcoholism), medical history (antibiotic allergy, diabetes mellitus [DM], and hypertension), clinical symptoms and signs, laboratory tests, and imaging results. Bacterial culture and sensitivity tests were performed using pus cultures or throat swabs. Pus was obtained by surgical incision of the abscess or ultrasound-guided aspiration. Clinical symptoms and signs comprised of categorical and continuous variables, including body temperature, heart rate, pharyngalgia, odynophagia, neck pain, neck swelling, dysphagia, trismus, hoarseness, and dyspnea. The primary region of infection was divided into the suprahyoid, infrahyoid, and retropharyngeal regions, as defined by Vieira et al. [11] Laboratory findings included neutrophil count, platelet count to lymphocyte count ratio (PLR), red blood cell count, hemoglobin, blood glucose, and albumin. Imaging results included multi-space involvement and gas formation. Other potential predictors were not considered if more than 5% of the values were missing. All categorical variables were defined as “yes” or “no” in the data analysis, except for the primary region of infection. All data were collected by two experienced clinicians.

### Metagenomic sequencing analysis

DNA was extracted using a QIAmp DNA Mini kit (QIAGEN, Hilden, Germany) according to the manufacturer’s instructions. DNA was used to construct a library using a Nextera XT DNA Library Prep Kit (Illumina, San Diego, CA) [12]. Library pools were then loaded on an Illumina NextSeq CN500 sequencer for 75 cycles of single-end sequencing of 20 million reads for each library, and sequence data were compared with those in a current bacterial and fungal database (NCBI; ftp://ftp.ncbi.nlm.nih.gov/genomes).

### Outcomes

Our primary goal was to analyze the distribution of bacteria in patients with DNSA based on disease severity and clinical characteristics. Patients with DNSA who had developed life-threatening complications were defined as severe cases, whereas those who did not develop life-threatening complications were defined as mild cases. Life-threatening complications included airway obstruction, descending mediastinitis, jugular thrombophlebitis, Lemierre’s syndrome, severe pneumonia, and sepsis. Patients who developed life-threatening complications before admission were excluded from the study. Since the use of antibiotics is based on the results of Gram-staining, we divided patients into Gram-positive group and Gram-negative group in the analysis of clinical characteristics of bacteriology. If the culture results revealed Gram-positive and negative strain coinfection, patients were included in the Gram-negative strain group because broad-spectrum antibiotics as an empiric treatment were required for these patients.

### Statistical analysis

Nominal variables are presented as frequencies (percentages) and continuous variables are presented as medians and interquartile ranges. The Mann–Whitney *U*, chi-square, and Fisher’s exact tests were used to compare variables between different groups.

We developed a prediction model for disease severity (mild/severe illness) based on a nomogram. Confounding predictors of the nomogram were adjusted using least absolute shrinkage and selection operator (LASSO) and logistic regression models. The odds ratios (ORs) and 95% confidence intervals (CIs) were calculated using the model. Finally, a web-based calculator (https://sysu.shinyapps.io/OnlineCalculator/) was constructed according to the nomogram. To assess the discriminative performance of the nomogram in the training and validation cohorts, the area under the curve (AUC) of the receiver operating characteristic (ROC) curve was measured. Hosmer–Lemeshow tests were used to assess the fitness of the model; an insignificant Hosmer–Lemeshow test statistic implies good model fitting. The GiViTI calibration was specifically designed to visually illustrate the relationship between the observed and predicted outcomes by fitting a polynomial function between the two, and statistically significant deviations occurred when the diagonal bisector line was not contained within the 95% CI.

Clinical characteristics were identified for the type of Gram reaction of the strain according to the baseline characteristics, clinical symptoms and signs, medical history, imaging findings, and laboratory tests in the mild disease group and the severe disease group, respectively. A *p*-value less than 0.05 was considered to indicate significance in each statistical analysis. Statistical analyses were performed using SPSS version 25.0 software (SPSS, Inc., Chicago, IL) and the R environment (version 4.0.5; R Foundation for Statistical Computing, Vienna, Austria).

## Results

### Characteristics of patients with DNSA

In this study, 433 patients with DNSA were consecutively recruited between January 1, 2015, and December 31, 2020, from nine medical centers in Guangdong Province. The median age of the patients was 51 years (interquartile range 38–61 years), and males accounted for 70.4%. Baseline characteristics, laboratory tests, and imaging findings are presented in Table 1. Overall, 92 (21.2%) patients developed the following life-threatening complications: descending mediastinitis (41.3%), airway obstruction (34.8%), sepsis (9.8%), jugular thrombophlebitis (7.6%), severe pneumonia (5.4%), and Lemierre’s syndrome (1.1%).

**Table 1:** Demographics and clinical characteristics of patients with DNSA

Predictors	All (n = 433)	Training (n = 359)	Life-threatening complications		*P* Value
			Yes (n = 77)	No (n = 282)	
Age, median (IQR), years	51.0 (38.0–61.0)	51.0 (37.0–61.0)	61.0 (53.0–67.5)	46.0 (35.0–58.0)	** < .001**
Sex, male, n (%)	305 (70.4)	246 (68.5)	53 (68.8)	193 (68.4)	.948
Antibiotic allergy, n (%)	24 (5.5)	22 (6.1)	7 (9.1)	15 (5.4)	.280
Smoking, n (%)	108 (24.9)	88 (24.5)	18 (23.4)	70 (24.8)	.794
Alcoholism, n (%)	39 (9.0)	31 (8.6)	9 (11.7)	22 (7.8)	.282
Body temperature, median (IQR), ℃	36.6 (36.4–36.9)	36.6 (36.4–36.9)	36.6 (36.5–37.0)	36.6 (36.4–36.8)	.338
Heart rate, median (IQR), bpm	87.0 (78.0–100.0)	87.0 (78.0–99.0)	92.0 (80.0–110.0)	86.0 (77.8–97.3)	**.002**
Diabetes mellitus, n (%)	113 (26.1)	96 (26.7)	31 (40.3)	65 (23.0)	**.002**
Hypertension, n (%)	65 (15.0)	52 (14.5)	18 (23.4)	34 (12.1)	**.012**
Multispace involvement, n (%)	213 (49.2)	176 (49.0)	69 (89.6)	107 (37.9)	** < .001**
Gas formation, n (%)	127 (29.3)	103 (28.7)	54 (70.1)	49 (17.4)	** < .001**
Initial onset of symptoms and signs					
Pharyngalgia, n (%)	293 (67.7)	238 (66.3)	57 (74.0)	181 (64.2)	.105
Odynophagia, n (%)	241 (55.7)	198 (55.2)	51 (66.2)	147 (52.1)	**.027**
Neck pain, n (%)	289 (66.7)	231 (64.3)	47 (61.0)	184 (65.2)	.494
Neck swelling, n (%)	256 (59.1)	210 (58.5)	45 (58.4)	165 (58.5)	.991
Dysphagia, n (%)	185 (42.7)	151 (42.1)	44 (57.1)	107 (37.9)	**.002**
Trismus, n (%)	94 (21.7)	74 (20.6)	13 (16.9)	61 (21.6)	.361
Hoarseness, n (%)	30 (6.9)	26 (7.2)	10 (13.0)	16 (5.7)	**.028**
Dyspnea, n (%)	63 (14.5)	50 (13.9)	32 (41.6)	18 (6.4)	** < .001**
Primary regions of infection					
Suprahyoid region, n (%)	295 (68.1)	244 (68.0)	56 (72.7)	188 (66.7)	.312
Infrahyoid region, n (%)	96 (22.2)	78 (21.7)	4 (5.2)	74 (26.2)	** < .001**
Retropharyngeal region, n (%)	42 (9.7)	37 (10.3)	17 (22.1)	20 (7.1)	** < .001**
Laboratory test					
NEUT, median (IQR), 10^9^/L	10.8 (7.7–14.5)	10.8 (7.5–14.5)	13.4 (9.8–17.5)	10.4 (7.2–13.9)	** < .001**
PLR, median (IQR)	181.4 (132.0–258.2)	183.3 (132.5–259.1)	235.4 (170.8–354.4)	173.4 (125.3–227.5)	** < .001**
RBC, median (IQR), 10^12^/L	4.6 (4.2–5.0)	4.6 (4.2–5.0)	4.5 (4.0–4.9)	4.6 (4.3–5.0)	**.007**
Hb, median (IQR), g/L	135.0 (123.0–147.0)	135.0 (123.0–146.0)	132.0 (118.0–144.5)	136.0 (124.8–148.0)	.071
Blood glucose, median (IQR), mmol/L	6.1 (5.1–9.5)	6.1 (5.1–9.4)	8.4 (6.4–12.1)	5.8 (5.0–8.1)	** < .001**
Albumin, median (IQR), g/L	38.1 (33.5–42.5)	38.2 (33.5–42.6)	31.8 (27.0–36.0)	39.2 (36.0–43.3)	** < .001**

Bold *P* values are statistically significant

DNSA, deep neck space abscess; IQR, interquartile range; bpm, beat per minute; NEUT, neutrophile count; PLR, platelet count to lymphocyte count ratio; RBC, red blood cell; Hb, hemoglobin

### Construction of disease severity prediction model

**Fig. 1:**
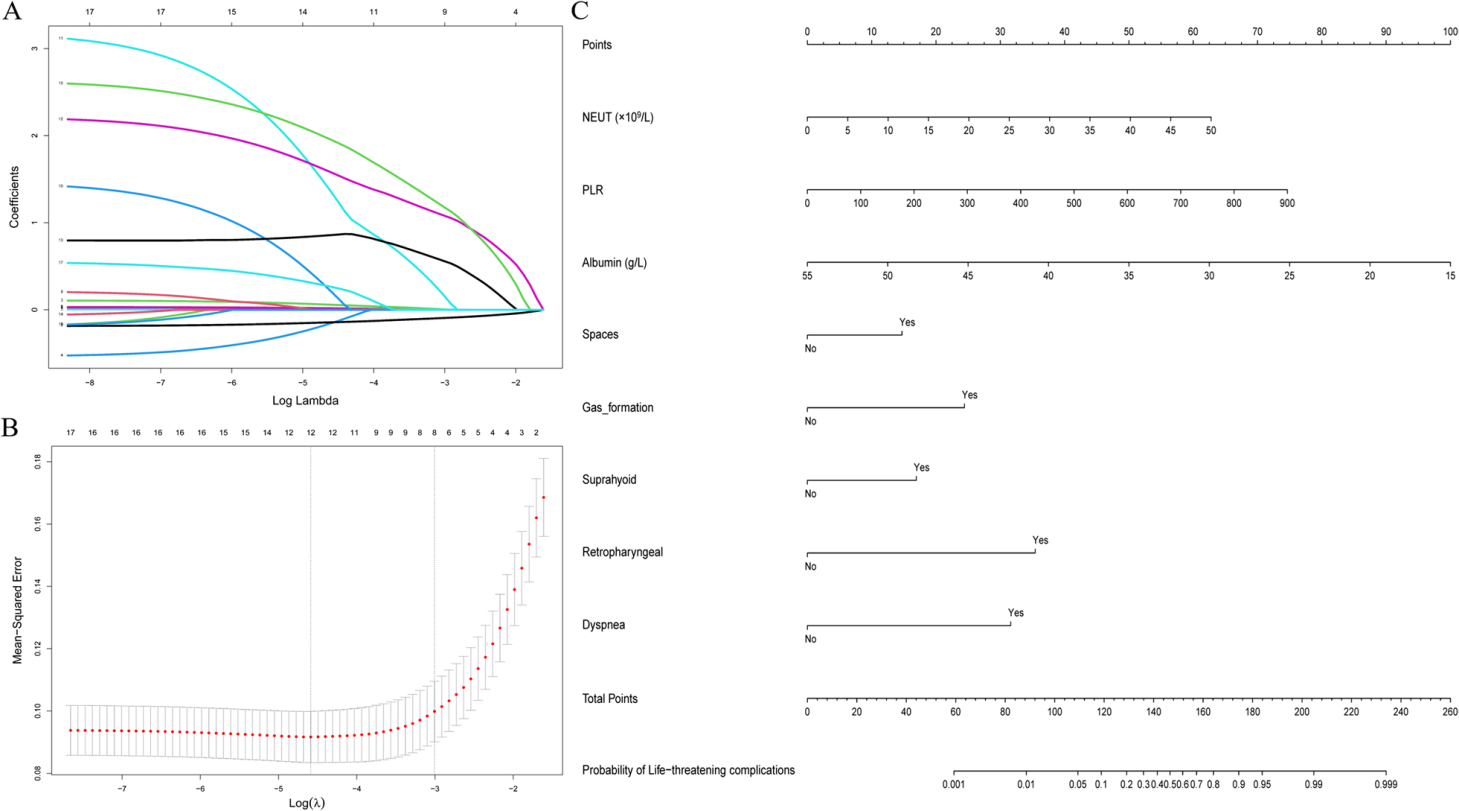
LASSO and Logistic Regression Models Used to Select Variables. **A** LASSO coefficient profiles of 17 clinical features. **B** Identification of the optimal penalization coefficient λ (0.054) in the LASSO regression model with tenfold cross-validation and 1 SE criterion. **C** Nomogram for assessing the risk of developing life-threatening complications in patients with DNSA. Abbreviations: LASSO, least absolute shrinkage, and selection operator; DNSA, deep neck space abscess; NEUT, neutrophil count; PLR, platelet count to lymphocyte count ratio; Spaces, multi-space involvement

In the training cohort, the predictors shown in Table 1 was assessed by univariate analyses. Seventeen predictors were significantly associated with severe illness. After LASSO regression selection, nine predictors remained as predictors of severe illness (Fig. 1A, B). Inclusion of these variables in a logistic regression model identified seven predictors that were independent significant predictors of severe illness and were included in the nomogram (Fig. 1C). These variables were multi-space involvement, gas formation, primary regions of infection, dyspnea, neutrophil count, PLR, and albumin level (Table 2).

**Table 2:** Multivariate analysis of life-threatening complications for patients with DNSA in the training cohort

Predictors	OR (95% CI)	*P* Value
Multispace involvement	3.20 (1.12–9.11)	.029
Gas formation	6.90 (2.92–16.28)	< .001
Regions of infection		
Infrahyoid	Reference	
Suprahyoid	3.82 (0.93–15.64)	.063
Retropharyngeal	16.46 (3.14–86.2)	< .001
Dyspnea	12.18 (4.29–34.60)	< .001
NEUT	1.10 (1.03–1.18)	.005
PLR	1.01 (1.00–1.01)	< .001
Albumin	0.82 (0.77–0.88)	< .001

DNSA, deep neck space abscess; NEUT, neutrophile count; PLR, platelet count to lymphocyte count ratio; RBC, red blood cell; Hb, hemoglobin; OR, odds ratio; CI, confidence interval

**Fig. 2:**
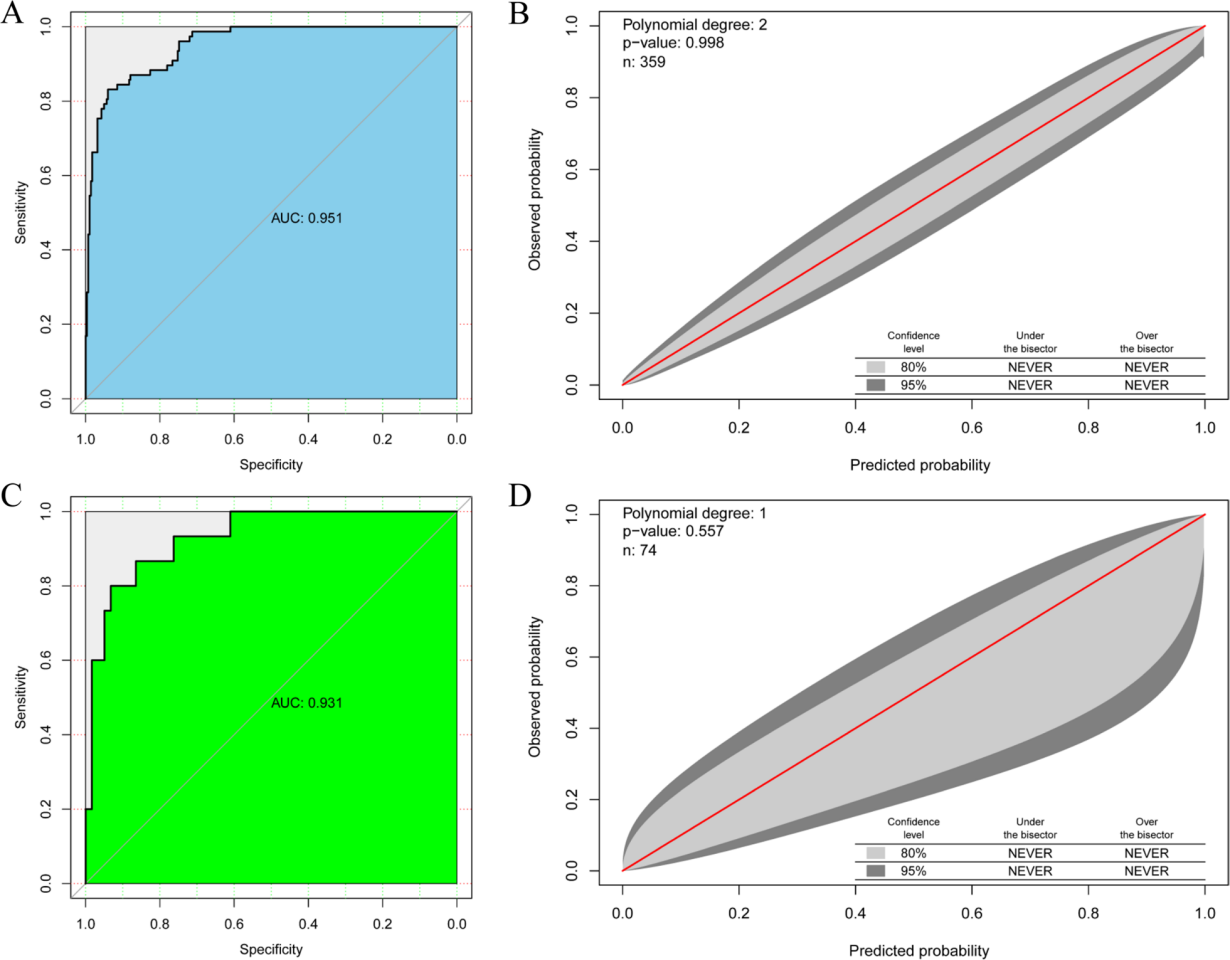
Performance for the Nomogram. Receiver operation characteristics curves (**A**) and GiViTI calibrations (**B**) in the training cohort. Receiver operation characteristics curves (**C**) and GiViTI calibrations (**D**) in the validation cohort

In internal validation, ROC analysis showed that the resulting model had good discrimination with an AUC of 0.951 (95%CI, 0.923–0.971) (Fig. 2A). The cut-off point was 139.9 (corresponding to a threshold probability of 37.0%). The Hosmer–Lemeshow test revealed no significance (*P* = 0.853), suggesting the good fit of the model. In addition, the GiViTI calibration plot graphically showed that the prediction and observation data agreed well for the training cohort (Fig. 2B). In the external validation cohort (Table 3), the model displayed good discrimination with an AUC of 0.931 (95%CI, 0.848–0.977) (Fig. 2C). Good model fit was also illustrated by the non-statistical significance obtained in the Hosmer–Lemeshow test (*P* = 0.471). Figure 2D shows the good overall calibration with the GiViTI calibration belt encompassing the entire bisector. Finally, we compiled the nomogram using a web-based calculator (https://sysu.shinyapps.io/OnlineCalculator/).

**Table 3:** Demographics and clinical characteristics of patients with DNSA in the validation cohort

Predictors	All (n = 74)	Life-threatening complications	
		Yes (n = 15)	No (n = 59)
Age, median, (IQR), years	53.0 (42.8–59.8)	58.0 (46.0–68.0)	52.0 (41.0.0–57.0)
Sex, male, n (%)	59 (79.7)	14 (93.3)	45 (76.3)
Multispace involvement, n (%)	37 (50.0)	15 (100.0)	22 (37.3)
Gas formation, n (%)	24 (32.4)	10 (66.7)	14 (23.7)
Dyspnea, n (%)	13 (17.6)	7 (46.7)	6 (10.2)
Primary regions of infection			
Suprahyoid region, n (%)	51 (68.9)	14 (93.3)	37 (62.7)
Infrahyoid region, n (%)	18 (24.3)	0 (0.0)	18 (30.5)
Retropharyngeal region, n (%)	5 (6.8)	1 (6.7)	4 (6.8)
Laboratory test			
NEUT, median, (IQR), 10^9^/L	11.3 (8.7–14.7)	13.0 (11.6–20.8)	10.2 (8.4–13.4)
PLR, median, (IQR)	172.5 (115.5–257.1)	231.6 (160.1–420.4)	157.4 (113.5–241.4)
Albumin, median, (IQR), g/L	38.0 (33.4–41.9)	34.6 (27.8–35.1)	39.4 (33.9–42.5)

DNSA, deep neck space abscess; IQR, interquartile range; NEUT, neutrophile count; PLR, platelet count to lymphocyte count ratio

### Clinical characteristics for the type of Gram reaction of strains in patients with mild disease

**Fig. 3:**
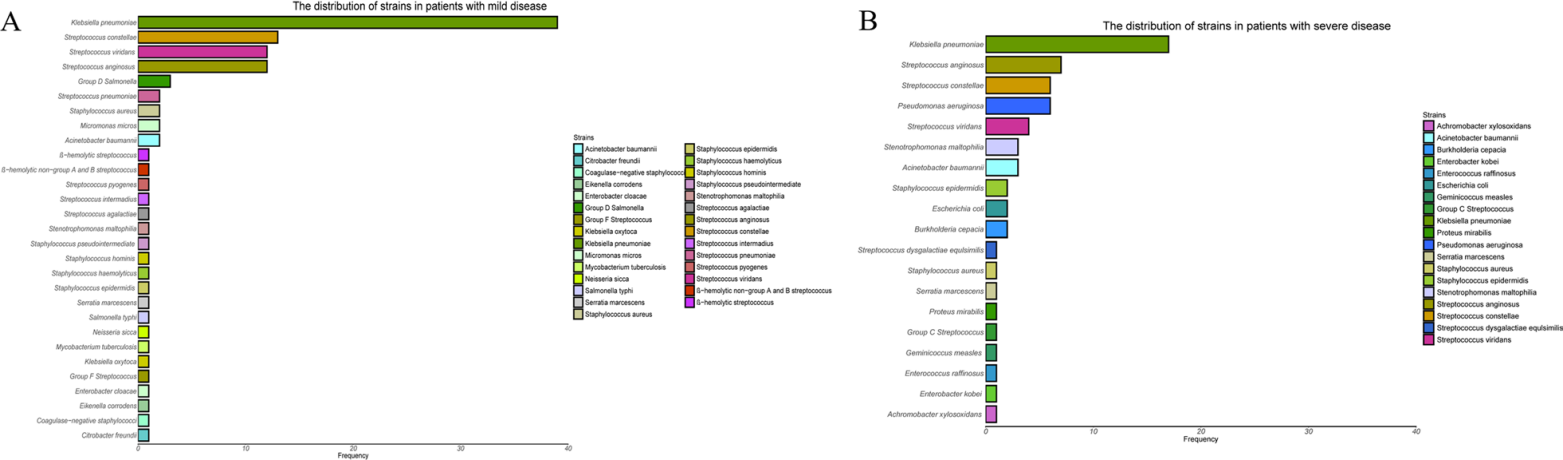
Distributions of Strains in Patients with Mild (**A**) and Severe (**B**) DNSA. DNSA, deep neck space abscess

In total, 341 patients were defined as mild cases. 107 (31.4%) of them were administered conservative treatment, and no pus was obtained. Cultures were obtained from the remaining 234 (68.6%) patients, and positive bacterial cultures were found in 101 (43.2%). The cultures of 49 (49%) patients showed Gram-positive strains, and the remaining (51%) showed Gram-negative strains. *Streptococcus anginosus*, *Streptococcus constellatus*, and *Streptococcus viridans* were the most common Gram-positive strains isolated, whereas *Klebsiella pneumoniae* was the most common Gram-negative strain isolated. Only one (1.0%) strain (*K. pneumoniae*) was found to produce extensive-spectrum β-lactamase. Figure 3A shows the distribution of strains isolated from patients with mild disease. Moreover, *Mycobacterium tuberculosis* was isolated from one patient.

**Fig. 4:**
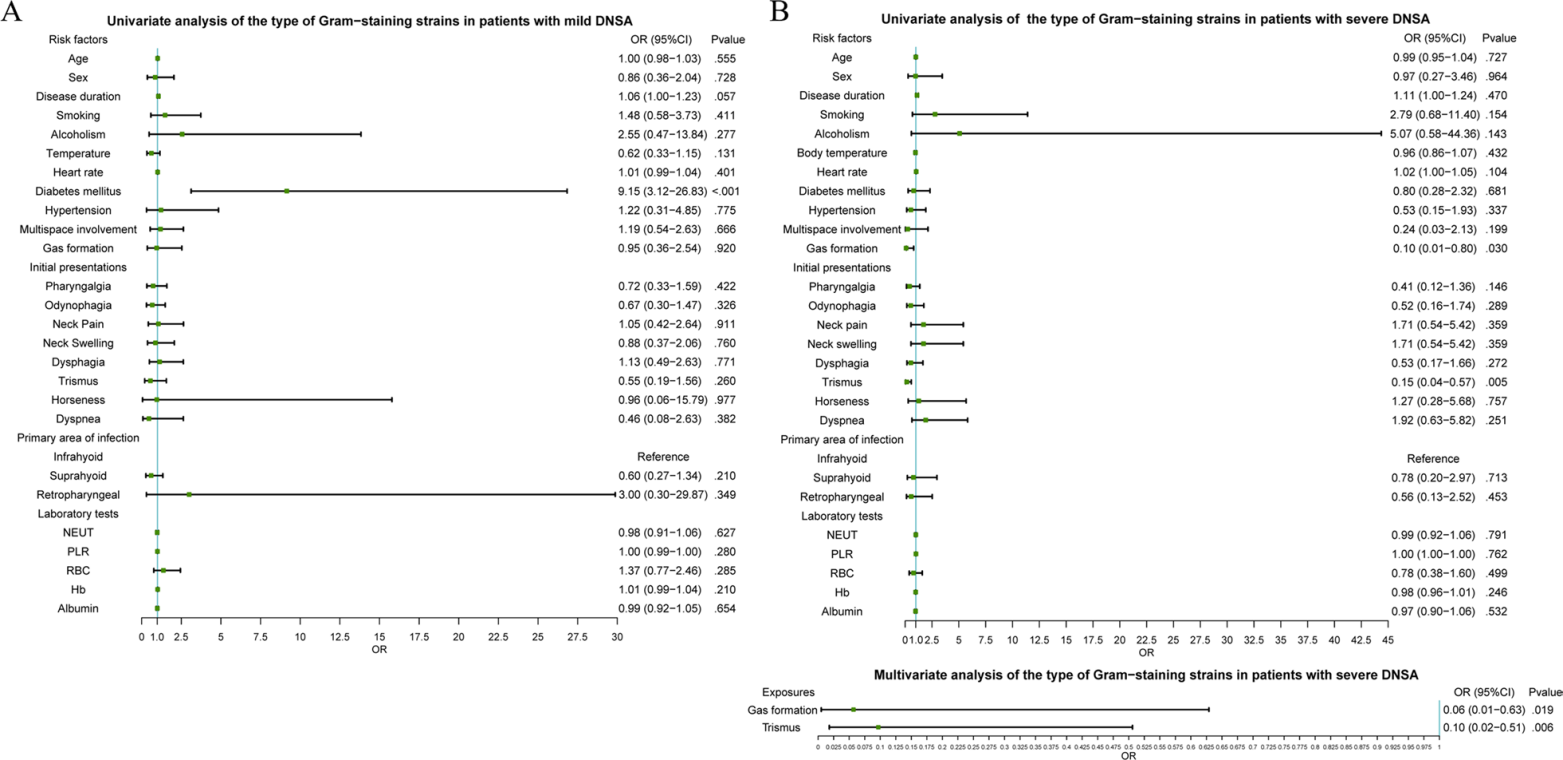
Univariate and Multivariate Analyses for the Type of Gram Reaction of Strains in Patients with Mild (**A**) and Severe (**B**) DNSA. DNSA, deep neck space abscess

**Fig. 5:**
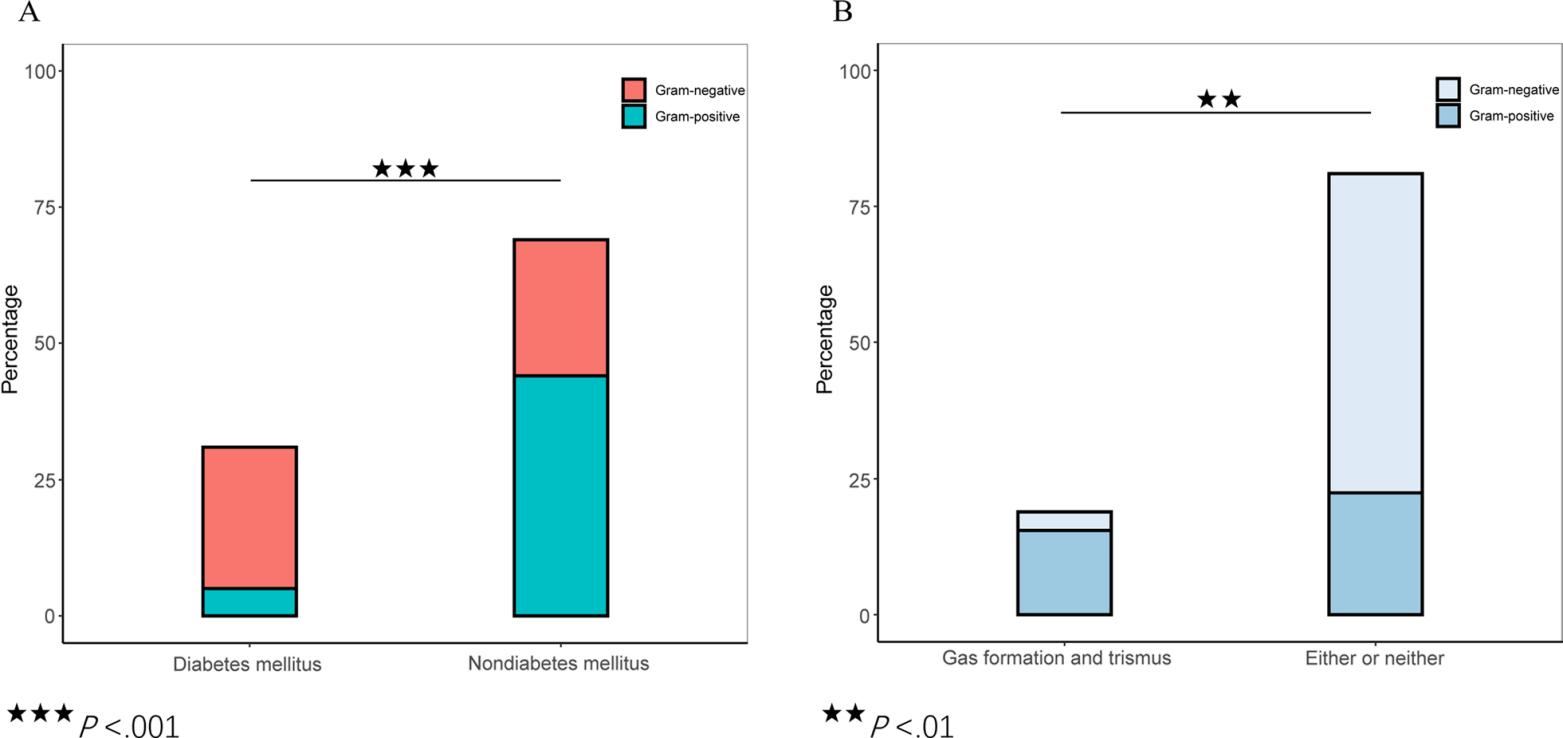
Differences of Gram-staining Strain Type based on clinical characteristics in Patients with Mild (**A**) and Severe (**B**) DNSA. DNSA, deep neck space abscess

Interestingly, we found that DM was a unique independent factor for Gram-negative strains (Fig. 4A), demonstrating that patients with mild disease and DM (OR = 9.15; 95%CI, 3.12–26.83) were likely to be infected by Gram-negative strains, whereas those without DM were likely to be infected by Gram-positive strains (Fig. 5A). This was an innovative discovery in that anti-infective treatment against Gram-negative bacteria should likely be used for patients with mild DNSA with DM.

### Clinical characteristics for the type of Gram reaction of strains in patients with severe disease

In patients with severe disease, pus cultures were positive in 58 (63.0%) patients and negative in 34 (37.0%) patients. Gram-positive strains accounted for 22 of the 58 (37.9%) positive cultures and the remaining 36 (62.1%) were Gram-negative bacteria. Figure 3B shows the distribution of strains in patients with severe disease. The top three Gram-negative strains were *K. pneumoniae*, *Stenotrophomonas maltophilia*, and *Pseudomonas aeruginosa*. The most common Gram-positive strains were *S. constellatus* and *S. anginosus*.

Gas formation (OR = 0.06; 95%CI, 0.01–0.63) and trismus (OR = 0.10; 95%CI, 0.02–0.51) significantly differed between the Gram-positive and Gram-negative groups in both univariate and multivariate analyses (Fig. 4B), illustrating that patient with severe disease and showing trismus and gas formation were more likely to be infected by Gram-positive strains; otherwise, they were likely to be infected by Gram-negative strains (Fig. 5B).

### Analysis of resistant strains and metagenomic sequencing

In patients with mild disease, the resistance rates of Gram-positive strains to penicillin, first/second-generation cephalosporins, and clindamycin were 12.2%, 6.1%, and 22.4%, respectively. The resistance rate of Gram-negative strains to third-generation cephalosporins, piperacillin, and quinolones (levofloxacin and moxifloxacin) were 7.8%, 3.9%, and 2.0%, respectively. In the severe disease group, three (5.2%) strains (two *K. pneumoniae* and one *Escherichia coli*) were found to produce extensive-spectrum β-lactamases, one strain was confirmed to be methicillin-resistant *Staphylococcus epidermidis*, and three (5.2%) strains (two *P. aeruginosa* and one *K. pneumoniae*) were found be carbapenem-resistant. The positivity rate of multidrug-resistant strains was higher in the severe disease group than in the mild disease group (12.1% vs. 1.0%, *P* < 0.001). All Gram-positive strains in the severe disease group were susceptible to amoxicillin-clavulanate and vancomycin, whereas the resistance rate of Gram-negative strains to piperacillin-tazobactam, cefoperazone-sulbactam, and carbapenem were 11.1%, 16.7, and 8.3%, respectively.

Metagenomic sequencing was performed on 34 specimens (22 and 12 patients with mild and severe disease, respectively). The positive rate obtained using this method was 88.2% (30/34), while the positive rate obtained using traditional methods was only 47.1% (16/34). The identification results of metagenomic sequencing analysis are roughly the same as those of conventional methods (Table 4), whereas the result of this advanced method was only available within 24 h. In addition, metagenomic sequencing has great advantages in detecting anaerobic bacteria. No anaerobic strains were detected in all specimens by conventional culture but detected in 25 (83.3%, 14 and 11 patients with mild and severe disease, respectively) samples by metagenomic sequencing. Thus, clinicians should consider the presence of anaerobic infection in patients with DNSA regardless of disease severity.

**Table 4:** The distribution of strains in bacterial culture and metagenomic sequencing

Strains	Cultures (n = 16)	Metagenomics (n = 30)
Aerobic Gram-positive strains		
Streptococcus constellation, n (%)	3 (18.8)	10 (33.3)
Streptococcus anginosus, n (%)	2 (12.5)	8 (26.7)
Streptococcus intermediate, n (%)	1 (6.3)	4 (13.3)
Staphylococcus aureus, n (%)	1 (6.3)	2 (6.7)
Aerobic Gram-negative strains		
Klebsiella pneumoniae, n (%)	5 (31.3)	5 (16.7)
Enterobacter cloacae, n (%)	2 (12.5)	0 (0)
Pseudomonas aeruginosa, n (%)	1 (6.3)	0 (0)
Burkholderia cepacia, n (%)	1 (6.3)	0 (0)
Anaerobic strains, n (%)	0	25 (83.3)

## Discussion

To the best of our knowledge, this is the first bacteriological analysis based on disease severity and clinical characteristics in patients with DNSA. We developed a new clinical prediction model based on disease severity in patients with DNSA, which demonstrated good discrimination and generalizability. This prediction model can help clinicians to early identify DNSA patients who are more likely to develop a severe illness. Moreover, we analyzed the distribution and predictive factors of the type of Gram-straining strains based on disease severity and clinical characteristics, and identified the existence of anaerobic infection through metagenomic sequencing analysis. These results can provide a new clinical basis for clinicians on how to reasonably select empiric antibiotics for patients with DNSA.

Prediction models for disease severity in patients with DNSA are lacking. In this study, a novel prediction model was constructed to assist clinicians in the early identification of patients who are likely to develop life-threatening complications. The model composed seven independent predictors that covered clinical symptoms and signs, imaging findings, and laboratory tests that can be used to systematically assess the severity of DNSA patients. Although previous studies have found that some inflammatory indicators are related to life-threatening complications in patients with DNSA, PLR is a new inflammatory marker that we first applied to the model for indicating disease severity [15–20]. In addition to inflammatory indicators, we also found that the primary regions of infection were strongly associated with disease severity. The primary region of infection originating from the suprahyoid region can cause Ludwig’s angina, and abscesses originating from the retropharyngeal space can easily spread downward and cause complications with mediastinal abscesses, while congenital branchial cleft fistulae mostly originate from the infrahyoid region, which are more limited in deep neck space and involve fewer life-threatening complications. Although the cut-off point of the model is relatively low, we still need to be vigilant about patients at high risk for developing critical complications. We divided the patients into low- (mild) and high-risk (severe) groups based on the cut-off point. Thus, this prediction model can provide a novel evaluation reference model for clinicians to distinguish the disease severity in patients with DNSA.

Previous studies have explored the distribution of bacteria in patients with DNSA, but none of them have performed a stratified analysis based on the disease severity and clinical characteristics. In our study, the proportion of Gram-positive and Gram-negative strains in patients with mild disease was approximately the same. Among them, the most common Gram-positive strain was *Streptococcus*, and the most common Gram-negative strain was *K. pneumoniae*. Interestingly, only DM was associated with the type of Gram-staining strain, indicating that patients with mild DNSA and DM were mainly infected by *K. pneumoniae*, which is consistent with the findings of a previous study [21]. Therefore, third-generation cephalosporins, piperacillin, quinolones can be recommended as empiric antibiotics to treat patients with mild DNSA and DM (Gram-negative strains), whereas penicillin and first/second-generation cephalosporins can be recommended as an empiric antibiotic in patients with mild DNSA without DM (Gram-positive strains). The low resistance rates to these antibiotics also support these findings.

Notably, more than half (62.1%) of patients with severe disease were infected by Gram-negative strains, and the overall multi-drug resistance rate reached 12.1%, which indicated that broad-spectrum antibiotics in combination with beta-lactamase inhibitors would be more in line with the bacteriological characteristics in patients with severe disease. Additionally, it is interesting that trismus and gas formation was significantly correlated with the type of Gram-positive strain in patients with severe disease. The most common cause of trismus is tonsillogenic or odontogenic infection in patients with DNSA, and the main pathogens of both are Gram-positive strains (e.g., *Streptococcus*) [22–24]. Gas formation is a sign of anaerobic pathogens (e.g., *Clostridium perfringens*, which is also a Gram-positive strain) and a higher complication rate [25, 26]. Poeschl et al. [27] reported that β-lactamase inhibitor combinations (amoxicillin-clavulanate) are more effective than penicillin for treating life-threatening complications such as mediastinitis, thoracic empyema, and septicemia. The isolates obtained from the severe disease group also showed a low resistance rate to amoxicillin-clavulanate, vancomycin, piperacillin-tazobactam, cefoperazone-sulbactam, and carbapenem. Therefore, according to the Gram reaction of strains mentioned above in patients with severe disease, amoxicillin-clavulanate or vancomycin can be selected as an empiric antibiotic in patients with severe disease exhibiting trismus and gas formation (Gram-positive strains), whereas piperacillin-tazobactam or cefoperazone-sulbactam can be selected as an empiric antibiotic for patients with severe disease who do not present the above symptoms (Gram-negative strains). According to the International Guidelines for the Management of Sepsis (2016), if the infection is poorly controlled and develops into sepsis, and the pathogen remains unidentified, the initial empiric antibiotics can be replaced with carbapenems [28]. In addition, it is worth noting that empiric antibiotic treatment should be deescalated to the narrowest effective agent once pathogen identification and sensitivities are established and/or adequate clinical improvement is noted.

Although bacterial culture is still the first-line detection method for identifying pathogens, the detection time required for metagenomic sequencing in our study was shorter (< 24 h) than that of bacterial culture, and the positive and accuracy rates were sufficient to outweigh the shortcomings of traditional methods. This advanced method can provide a reference for rapid pathogen diagnosis and allow the use of narrower-spectrum antibiotics in patients with DNSA. Moreover, metagenomic sequencing can also be used for subtyping the bacterial species and monitoring hospital outbreaks to support infection control and public health surveillance efforts [29]. Besides, the positivity rate of anaerobic culture was extremely low (9.5%) in both patients with mild and severe cases. This may be related to the harsh culture conditions used to grow anaerobic strains. However, the results of metagenomic sequencing showed that anaerobic strains were mixed in the pus, with a positivity rate as high as 83.3%. Many studies have confirmed that DNSA most commonly involves mixed infections of aerobic and anaerobic strains [2, 30–32]. Therefore, treatment of anaerobic infections should be considered in patients with DNSA regardless of the disease severity.

This study had several limitations. First, DNSA is a relatively rare infectious disease; we observed a low positivity rate in bacterial culture, resulting in a relatively small sample size. Second, different regions across the country showed different distributions of strains and drug resistance, and the results may be biased for cases outside of the Pearl River Delta region in Guangdong Province. Finally, we did not analyze prognosis, as this study was focused on bacteriology. With the support of this bacteriological evidence, it can provide a scientific basis for us to carry out the clinical trial of antibiotic treatment in patients with DNSA in the next research phase.

## Conclusion

In this study, we developed a prediction model and web-based calculator to estimate the risk of severe illness development in patients with DNSA. Estimating disease severity in patients with DNSA is a prerequisite for the rational selection of empiric antibiotics. The Gram reaction of the strain depends on the presence of DM in patients with mild DNSA, whereas in patients with severe DNSA, it mainly depends on the presence of trismus and gas formation. Multi-drug resistant strains were more common in patients with severe disease. Metagenomic sequencing can improve the positive detection rate of pathogenic bacteria and accurately detect the existence of anaerobic bacteria. Regardless of disease severity, it is necessary to consider the treatment of anaerobic infections. Empiric antibiotics must be modified after the results of bacterial culture and susceptibility tests are available.

## Data Availability

The datasets generated and/or analysed during the current study are available in the NCBI SRA repository (https://dataview.ncbi.nlm.nih.gov/object/PRJNA810198?reviewer=3hf38iat1ruji12tfur8b263po).

## Abbreviations

DNSA: Deep neck space abscess
ASP: Antimicrobial stewardship program
DM: Diabetes mellitus
PLR: Platelet count to lymphocyte count ratio
LASSO: Least absolute shrinkage and selection operator
OR: Odds ratio
CI: Confidence interval
AUC: Area under the curve
ROC: Receiver operating characteristic
NEUT: Neutrophil count
RBC: Red blood cell
Hb: Hemoglobin
IQR: Interquartile range

## Code availability

The R codes used for processing the data during the current study are available from the corresponding author on reasonable request.
